# Supplementary material for: The genome sequence of the parsley Cheilosia, Cheilosia pagana (Meigen, 1822)
Source: Wellcome Open Res. 2024 Feb 19;9:54. [Version 1] doi: 10.12688/wellcomeopenres.20627.1 (PMC11134149; doi:10.12688/wellcomeopenres.20627.1)
Supplement: Peer review history

# Reviewer report: Reviewer response for version 1

**DOI:** 10.21956/wellcomeopenres.22830.r82350

**Published:** 2024-05-28

**Authors:** Charles Plessy

**Affiliations:** 1 Okinawa Institute of Science and Technology Graduate University (OIST), Okinawa, Japan

The article is clear and follows the same pattern as the other works published here by the authors on other *Cheilosia* flies, which makes it easy to assess.

 In this assembly and some other *Cheilosia* assemblies as well, chromosome 1 is noticeably longer than the others.  Hi-C data shows a repeated region near its center.  Can the authors comment on whether they think it is a centromere, and if the one of chr1 is much larger than the others, or if centromeres are just missing from the other assembled chromosomes?

Are sufficient details of methods and materials provided to allow replication by others?

Yes

Is the rationale for creating the dataset(s) clearly described?

Yes

Are the datasets clearly presented in a useable and accessible format?

Yes

Are the protocols appropriate and is the work technically sound?

Yes

Reviewer Expertise:

Pairwise genome alignment

I confirm that I have read this submission and believe that I have an appropriate level of expertise to confirm that it is of an acceptable scientific standard.

# Reviewer report: Reviewer response for version 1

**DOI:** 10.21956/wellcomeopenres.22830.r82347

**Published:** 2024-05-16

**Authors:** Adrián Casanova Chiclana

**Affiliations:** 1 Universidade de Santiago de Compostela, Lugo, Spain

In general, this article is clear and well-conducted. I would make some suggestions across sections.

**Keywords**

 I would recommend using "Syrphidae" (the Family) instead of "Diptera" (the Order). The former encompasses around 6K described species, the second more than 100K. Maybe using "Syrphidae" could better contextualise future readers.

**Background**

 It might be adequate to add the sex determination system of this species explicitly (in one sentence) within the first paragraph. I understood this species follows an XY sex-determination system, like other congeneric species (Crowley et al., 2023).

 I would add some examples of pollinated plants. It might be interesting to link with The Database of Pollinator Interactions (DoPI ;https://www.sussex.ac.uk/lifesci/ebe/dopi/), which contains some plants pollinated by this species.

 I would emphasize a bit more that this species participates in pollinator webs. As it is commented on the Center for Plant Conservation website (see https://saveplants.org/pollinator-search/) we cannot conserve plants without knowledge about (and conservation of) their pollinators.

**Methods**

 To increase reproducibility, I highly recommend creating a Supplementary Material or a GitHub page containing all the commands with the selected parameters/options. This will be very useful for the whole scientific community.

 I strongly recommend integrating the following sentence better within the main text: "Table 3 contains a list of relevant software tool versions and sources." It could be at the beginning of the "Genome assembly, curation and evaluation" subsection.

Are sufficient details of methods and materials provided to allow replication by others?

Partly

Is the rationale for creating the dataset(s) clearly described?

Yes

Are the datasets clearly presented in a useable and accessible format?

Yes

Are the protocols appropriate and is the work technically sound?

Yes

Reviewer Expertise:

Population genomics, Conservation genomics, Bioinformatics, Aquaculture

I confirm that I have read this submission and believe that I have an appropriate level of expertise to confirm that it is of an acceptable scientific standard.

## References

[1] : The genome sequence of the Figwort Cheilosia, Cheilosia variabilis (Panzer, 1798). *Wellcome Open Res* .2023;8: 10.12688/wellcomeopenres.19851.1 377 10.12688/wellcomeopenres.19851.1 37981983 PMC10654491
